# Comparison of Mycotic Keratitis with Nonmycotic Keratitis: An Epidemiological Study

**DOI:** 10.1155/2014/254302

**Published:** 2014-12-07

**Authors:** Mohammad M. Khater, Nehal S. Shehab, Anwar S. El-Badry

**Affiliations:** ^1^Ophthalmology Department, Tanta University, Gharbeya Governorate, El Geish Street, Tanta 31111, Egypt; ^2^Public Health Department, Tanta University, Gharbeya Governorate, El Geish Street, Tanta 31111, Egypt; ^3^Botany Department, Faculty of Science, Tanta University, Gharbeya Governorate, El Geish Street, Tanta 31111, Egypt

## Abstract

*Purpose*. This work aims to study the problems encountered with and the different epidemiological features of patients with fungal keratitis.* Patients and Methods*. All cases with keratitis attending the Outpatient Clinic of Ophthalmology Department at Tanta University Hospital during three years from the first of January 2011 to the end of December 2013 were selected and carefully examined and cases with mycotic keratitis were further examined and investigated.* Results*. From 66303 attendants during this period with different complaints, there were 361 cases (0.54%) with mycotic keratitis and 473 cases (0.71%) of nonmycotic origin. Mycotic keratitis is common between 40 and 60 years, more in farmers (64%), families with large number and large crowding index, rural than urban residence, and patients with outdoor water sources and insanitary sewage disposal. Positive fungal cultures were obtained in 84.5% and were negative in 15.5% of cases in spite of their typical clinical findings for diagnosis and their improvement with antifungal therapy.* Conclusion*. Mycotic keratitis is more frequent in farmers, rural areas, outdoor water supply, insanitary sewage disposal, and patients preceded with organic trauma. Atypical clinical findings were found in some cases and not all cases improved with specific antifungal therapy.

## 1. Introduction

Mycotic keratitis is a severe problem in most of the developing countries whereas specific antifungal agents are expensive and commercially unavailable as ready compounds for topical ocular use. It is endemic and this is favored by certain ecological factors that already exist in those communities. Other problems like difficulties in its clinical diagnosis, laboratory results, and treatment are also incriminated [1].

Mycotic keratitis is an important cause of corneal morbidity, scarring, and blindness caused by fungal invasion through corneal epithelium. The disease was found to be more common among agricultural communities than others. According to predisposing factors, clinical features, and response to treatment, fungal corneal pathogens were divided into filamentous fungi, yeast, and yeast like and dimorphic fungi. The most common filamentous fungi causing keratomycosis are* Aspergillus*,* Fusarium*,* Curvularia*,* Alternaria,* and* Cladosporium*, while* Candida albicans* is the most common form of yeasts [2, 3].

Mycotic keratitis probably arises as a result of an interaction between different agent factors as invasive ability and toxicity of fungal strains and host factors due to local or systemic causes. The invasiveness of fungal strains is aided by certain properties such as the capacity of fungus to adhere to the cells and to produce enzymes and toxins that destroy anatomical defenses [4, 5].

Fungi especially filamentous species rarely infect healthy cornea spontaneously and are usually predisposed by trauma with vegetable or organic matter. On the other hand* Candida albicans* is a common infection in compromised or immune-suppressed cornea [6, 7].

Identification of pathogenic corneal fungi is performed either by microscopic stained corneal scrapes or by their cultural features. Other methods for fungus identification depend on the types of enzymes produced, immune diffusion, electrophoresis, and ELISA. Confocal microscopy also plays a role in diagnosis of fungal keratitis [8–10].

The three major groups of antifungal agents are as follows:

1. polyenes, as amphotericin B and nystatin,
2. azoles, as itraconazole and fluconazole,
3. pyrimidines, as flucytosine [11, 12].

## 2. Patients and Methods

The study was conducted from the first of January 2011 to the end of December 2013 at the Outpatient Clinic of Ophthalmology Department in Tanta University Hospital.

A total number of 66303 patients with different ophthalmological complaints were examined during that period. All patients with corneal lesions were selected and carefully examined.

Our target included those cases diagnosed clinically as fungal keratitis; they were 361 cases during that period. Clinical diagnosis of mycotic keratitis was dependent on some clinical criteria including the following:

- thick area of keratitis,
- thick hypopyon “coagulum” with or without level,
- immune rings,
- satellite lesions,
- stromal infiltrate with feathery edge,
- healing usually with dense leucoma with large solitary blood vessel,
- area of epithelial defect usually smaller than stromal infiltration.

The other group included all cases of nonmycotic keratitis who attended the clinic during the same period. They were 473 cases of both bacterial and viral origin.

All cases of mycotic and nonmycotic keratitis were submitted to a complete ophthalmological examination by the slit lamp using direct oblique, scleral scatter, and retroillumination techniques. Corneal staining with fluorescein and rose bengal was done.

A questionnaire sheet was designed and filled by the researchers for cases and control including the following.

(1) Biological and sociodemographic data: age, sex, occupation, family size, crowding index (family members/number of rooms), residence, source of water whether indoor or outdoor, and sewage disposal whether pipes system or conservancy system.

(2) History taking for the following: some risk factors such as recent drug intake (prolonged antibiotics and local or systemic corticosteroids), corneal trauma whether organic or nonorganic, systemic diseases (diabetes mellitus, tuberculosis, and cancer), ocular surgery, local eye diseases such as glaucoma, and the time of onset of complaints till examination.

(3) Laboratory investigations: all cases diagnosed clinically as fungal keratitis were subjected to the following:

- *Sampling:* Under surface anesthesia, the active edge and the bed of ulcer were scraped using platinum spatula or sterile surgical blade (number 15) with the aid of slit lamp biomicroscopy or surgical microscope to ensure that adequate corneal material was obtained and to avoid corneal perforation. Ulcers were very soft, sticky, and fucoid in consistency. So disposable calcium alginate cotton tipped sterile applicators were used for those cases.
- *Isolation media:* Sabouraud dextrose agar was prepared with 0.05% chloramphenicol, autoclaved, and spread on sterile Petri plates. Plates were incubated at room temperature; fungal growth was identified and then sensitivity tests were carried out in vitro by using different antifungal agents. According to the results of sensitivity tests, the appropriate antifungal agent was used as topical drops, subconjunctival injection, and intracameral wash and even systemically if needed depending on the severity of the case.

The treatment regimen used for all cases was as follows:

- amphotericin B (2 mg/mL) (Fungizone; Bristol-Myers Squibb) was used topically till the results of culture appeared;
- when the culture results appeared, the appropriate antifungal agent was used;
- the antifungal drugs prescribed included amphotericin B 0.15% (Fungizone; Bristol-Myers Squibb) prepared as fortified drops, fluconazole 0.2% (Diflucan; Pfizer) taken directly in eye dropper from the vial, natamycin 5% (Natamet; Sun Pharmaceutical Industries), and itraconazole 1% (Itral; Jawa Pharmaceuticals).

*Adjunctive Therapy*

- Atropine sulphate (Isopto Atropine; Alcon) eye drops, 3 times/day.
- Gatifloxacin (Zimmer; Allergan) eye drops, 5 times/day,
  - weak corticosteroid drops of fluorometholone (FML; Allergan) twice daily added if there is immune ring or associated uveitis.

For cases not responding to treatment within the first week, a debridement of superficial corneal layers was done to enhance good penetration of antifungal therapy.

### 2.1. Statistics

Statistical presentation and analysis of the present study were conducted, using the mean, standard deviation, chi square, and *t*-test by SPSS V.20.

## 3. Results

A total number of 66303 patients attended the Outpatient Clinic of Ophthalmology Department in Tanta University during the period in which the study was conducted. Our target was 361 patients with mycotic keratitis representing 0.54% of the total attendants, while those with nonmycotic keratitis accounted for 473 cases representing 0.71% of the total attendants of the clinic during that period.

### 3.1. Age and Sex

- The mean age for cases of mycotic keratitis was 49.18 ± 2.28 years, while mean age for the nonmycotic group was 50.88 ± 2.60 years.
- Table 1 shows that males in patients of mycotic keratitis were affected about twice more than females. They were 238 males accounting for 65.9% and 123 females representing 34.1%, respectively. The same was found with the nonmycotic keratitis group; they were 66.6% males and 33.4% females. However the difference was statistically insignificant between mycotic and nonmycotic groups as regards the sex (*χ* ^2^ = 6.4, *P* = 0.739).
- 52.4% of cases of mycotic keratitis were at the age group of 40–60 years while it was recorded that 51.4% of cases of nonmycotic keratitis were at the same age group. The difference between mycotic and nonmycotic keratitis was statistically not significant regarding the different age groups (*χ* ^2^ = 6.21, *P* = 0.258).

### 3.2. Sociodemographic Indicators

#### 3.2.1. Occupation

As shown in Table 2 farmers (unskilled workers) represented the majority of cases (68.4%) in mycotic keratitis group while they represented only 50.3% in the nonmycotic keratitis group. Professionals and semiprofessionals represented only 2.2% of the mycotic group compared to 19.9% among the nonmycotic group. Housewives were more among the mycotic cases than the nonmycotic cases; they were 20% and 13.3%, respectively. The difference was statistically significant between cases of mycotic and nonmycotic keratitis as regards their occupations (*χ* ^2^ = 21.41, *P* = 0.001).

#### 3.2.2. Family Size

As shown in Table 3, more than one-third of cases of mycotic keratitis were of large family size (>6 persons) compared to the nonmycotic group (38.2% and 20.3%), respectively, while the percentage of cases with small family size (<4 persons) was less than the nonmycotic group; they were 20% and 36.6%, respectively. The difference was statistically significant (*χ* ^2^ = 10.67, *P* = 0.004).

#### 3.2.3. Crowding Index

Table 3 showed that 67.3% of cases with mycotic keratitis lived in overcrowded houses with crowding index >2. This was more than that of nonmycotic keratitis (55.2%). There was no significant statistical difference between mycotic and nonmycotic keratitis regarding the crowding index (*χ* ^2^ = 3.03, *P* = 0.081).

#### 3.2.4. Residence

Table 4 showed that in cases of mycotic keratitis, 64.3% were rural residents compared to 56.2% among the nonmycotic group. The difference was statistically not significant between mycotic and nonmycotic groups (*χ* ^2^ = 1.55, *P* = 0.212).

#### 3.2.5. Water Source

As shown in Table 4, outdoor water supply represented about three-quarters (72.9%) of the houses of mycotic cases and more than half of those of cases with nonmycotic keratitis (58.1%). The difference was statistically significant (*χ* ^2^ = 4.95, *P* = 0.025).

#### 3.2.6. Sewage Disposal

Table 4 showed that in cases of mycotic keratitis only 22.7% of patients had pipes system of sewage disposal in their houses compared to 45% in the houses of nonmycotic group with a significant statistical difference (*χ* ^2^ = 10.78, *P* = 0.001).

### 3.3. Time between the Onset of Complaints and Examination

From Table 5, it was found that among the mycotic group 61.5% of cases came within the first week from the onset of complaints while about a quarter (22.4%) of cases came for ocular examination from 14 days up to 30 days. In nonmycotic corneal ulcers the majority (85%) came within the first week of complaints and no one came after 14 days. The difference between the two groups was statistically significant as regards the different periods of time (*χ* ^2^ = 25.63, *P* = 0.3).

### 3.4. Laboratory Results

**Figure 1:**
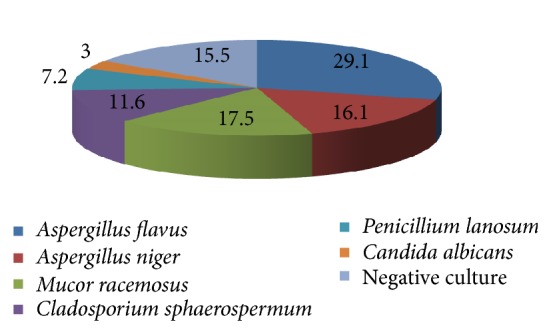
Types of fungal infections among mycotic keratitis cases.

Table 6 and Figure 1 revealed that* Aspergillus* species constituted the majority of cases with positive cultures:* Aspergillus flavus* in 29.1% of cases and* Aspergillus niger* in 16.1%, and the least frequent species was* Candida albicans* (3%). Negative cultures occurred in 15.5% of cases in spite of their improvement with antifungal therapy and their typical clinical findings for diagnosis.

### 3.5. Risk Factors

Table 7 showed that trauma either organic or nonorganic was the most frequent risk factor for both of mycotic (58.4%) and nonmycotic (75%) groups. Organic ocular trauma was the most frequent risk factor in mycotic keratitis (38.2%) while nonorganic trauma was the most risky for the nonmycotic group (45%). Other risk factors included ocular surgery which represented 32.7% of mycotic cases compared to 0% among nonmycotic group. Prolonged local antibiotics therapy was risky for 6.1% of mycotic keratitis compared to 0% among nonmycotic group. Systemic diseases represented the least frequent risk for mycotic keratitis (2.2% of the cases) while it was 10.4% in nonmycotic keratitis. All cases of mycotic keratitis that had a history of ocular surgery had a mild course of corticosteroid therapy except in 22 cases that had a prolonged heavy course.

### 3.6. Fate and Complications

**Figure 2:**
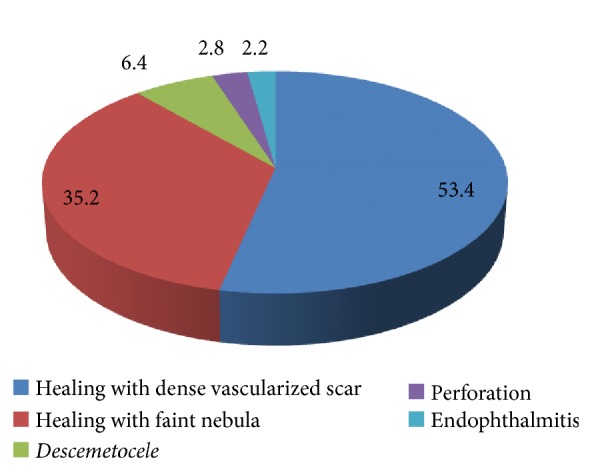
Fate and complications of mycotic cases.

Among the mycotic keratitis cases, 127 cases (35.2%) showed healing with faint nebulae, 193 cases (53.4%) with dense vascularized leucoma, 23 cases with descemetocele (6.4%), and 10 cases (2.8%) with perforation, and 8 cases (2.2%) ended by endophthalmitis as shown in Figure 2.

## 4. Discussion

Corneal infections including fungal types are responsible for about 50% of corneal scarring in the developing world which is a significant cause of blindness. Mycotic keratitis is a growing health problem in the developing countries representing from one-third up to 56% of total ocular infections. World Health Organization reported that about 10 million all over the world were blind due to corneal infections in its program for prevention of blindness (global initiative vision by the year 2020) [13, 14].

### 4.1. Age and Sex

In the present study, about half the cases of mycotic keratitis were at the age group from 40 to 60 years, and males were about two times more affected than females. These findings were close to that of Baradkar and others in 2008 [15] in their study in India whereas they concluded that fungal keratitis affected males three times more than females and were more frequent in the age group from 20 to 50 years. Similar results were obtained from other studies [11, 17]. Males are more affected because of their outdoor activity and movement in the surrounding environment which expose them to different risks such as corneal trauma. There were no significant statistical differences between cases of mycotic keratitis and those of nonmycotic keratitis as regards the age groups (*χ* ^2^ = 6.21, *P* = 0.258) and sex (*χ* ^2^ = 6.4, *P* = 0.739).

### 4.2. Sociodemographic Indicators

(i) In this study, mycotic keratitis affected farmers (unskilled workers) more than other occupations in 68.4% of the cases followed by housewives in 20%. These findings were consistent with Moharram and his colleagues in 1999 [16] in their study in Assiut where 47% of cases of fungal keratitis were farmers and 20% were housewives. Farming was the main job for most of cases of fungal keratitis in other studies [11, 15–17]. Most of fungal species such as* Aspergillus*,* Penicillium*,* Mucorrhacimosis* species, and* Cladosporium* are soil fungi, so they contaminate vegetables and fruits. Farmers are usually exposed to trauma by organic matter (such as dried rice stems or maize) which facilitates invasion of the cornea by the fungi. There was a significant statistical difference between cases of mycotic keratitis and the nonmycotic keratitis regarding their occupations (*χ* ^2^ = 21.41, *P* = 0.001). Farmers were more recorded among cases while professionals and semiprofessionals were more recorded among the control. Professionals might be exposed to some risk factors such as the nonorganic trauma but rarely exposed to organic trauma which is associated mostly with fungal contamination. In this study organic trauma was the most risky factor for fungal keratitis while nonorganic trauma was the most risky for the control group and this might explain the former finding.

(ii) This study reported that cases of mycotic keratitis belonged to large families (4–6 persons in 41.8% and more than 6 persons in 38.2%), with a high crowding index (67.3% of cases with crowding index >2), and about two-thirds (in 64.3%) of them were rural. There was a statistically significant difference between mycotic and nonmycotic cases as regards their family size (larger families among mycotic keratitis cases) (*χ* ^2^ = 10.67, *P* = 0.004). There were insignificant statistical differences between both groups regarding crowding index (*χ* ^2^ = 3.03, *P* = 0.081) and rural residence (*χ* ^2^ = 1.55, *P* = 0.212). However in both of mycotic and nonmycotic keratitis the percentage of the disease was higher in large families, with a high crowding index and rural residence. These were explained by the unsound health behavior and lack of health awareness especially in those living in overcrowded houses with bad sanitation. These factors facilitated infections especially with morbid corneas. These findings were in agreement with other studies [5, 18, 19] which reported that fungal and microbial keratitis associated with low socioeconomic status and rural residence.

(iii) As regards water sources, the outdoor water supply accounted for 72.9% of houses of mycotic cases compared to 58.1% among the nonmycotic cases with significant statistical difference (*χ* ^2^ = 4.95, *P* = 0.025). Concerning sewage disposal, more than three-quarters of houses of mycotic keratitis (77.3%) were by conservancy system compared to 55% of the houses in the nonmycotic cases with a significant statistical difference (*χ* ^2^ = 10.78, *P* = 0.001).

(iv) In conclusion, it was revealed that cases of mycotic keratitis lived in more deteriorated environment than the nonmycotic group. This was consistent with Gopinathan and his colleagues who reported in 2009 that cases of mycotic keratitis lived in poor insanitary environment [5]. Rautaraya in 2011 reported that eye washing with contaminated water could cause mycotic and nonmycotic keratitis [11]. Outdoor water and insanitary sewage disposal systems could affect personal hygiene and self-care with poor sanitation of houses together with spread of flies and insects that transmit organisms easily to healthy or unhealthy corneas.

### 4.3. Time of Onset of Complaints

In this study, 61.5% of cases of mycotic keratitis came within the first week of their onset of complaints to seek medical care compared to the majority (85%) in the nonmycotic group. About a quarter of cases of mycotic keratitis (22.4%) came to seek medical care after two weeks from onset of complaints compared to no one in the nonmycotic group. The difference in mean time between both groups was statistically significant (*χ* ^2^ = 25.63, *P* = 0.3). This might be explained by the following. First, some patients developed mycotic keratitis after ocular surgery and thought that their complaints were a normal sequence of the surgery, so they delayed seeking medical care. Second, some other patients were posttraumatic and thought that the symptoms were a normal sequence of trauma. Lastly, some patients were treated by mistake at first as nonmycotic keratitis till they were managed as fungal keratitis.

### 4.4. Laboratory Findings

(i) It was found that 84.5% of patients were positive, while 15.5% were negative in spite of their typical clinical findings and their improvement with antifungal therapy. These may be explained by some fungi present in deep stroma and so could not be obtained if superficial scraping was done or if scraping was obtained only from one area. These findings were in consistence with Rautaraya who reported that 25.4% of their patients showed negative fungal growth in spite of their typical clinical findings for microbial keratitis [11].

(ii) For the type of fungusisolated, it was found that* Aspergillus* species were the most common (45.2%), either* flavus* (29.1%) or* niger* (16.1%). These results were in agreement with Rautaraya and his colleagues in India who demonstrated that* Aspergillus* species constituted the majority (27.9%) of fungal growth in their study [11]. Another study in Mumbai in India demonstrated that* Aspergillus* species occurred in 17.6% [15]. Gopinathan reported in his study that* Aspergillus* species were isolated in 28.9% of cases [5]. These findings may be explained by the more spread of* Aspergillus* species in the environment specially spores which can survive hot and dry weather for long time.* Aspergillus* species existed in the soil and animal skin, so they were more frequent among the farmers in this study.

(iii) Among* Aspergillus* species it was found that* Aspergillus flavus* constituted 29.1% and* Aspergillus niger* 16.1%. This was consistent with the findings of Rautaraya study that demonstrated 15.8% for* Aspergillus flavus* and 12.3% for other* Aspergillus* species [11].

(iv)* Candida* species were the least frequent type (only in 3%); that is in agreement with what was reported by Rautaraya and others that* Candida* species were the least common in their study (0.9%) [11].

Our results revealed that, among a total number of 66303 cases examined during the time of the study, mycotic keratitis occurred among 0.54% of cases and the nonmycotic keratitis in 0.71%.

A study that was done from February 1991 to June 2001 by Gopinathan and others in India demonstrated 5897 cases of suspected infectious keratitis; 3563 (60.4%) were culture proven (bacterial: 1849, 51.9%; fungal: 1360, 38.2%; Acanthamoeba: 86, 2.4%; mixed: 268, 7.5%). Among fungal cases* Aspergillus* species were responsible for 28.9% of cases,* Fusarium* in 35.6%, dematiaceous fungi in 19.3%, other hyaline fungi in 15.4%, and* Candida* only in 0.8% [5].

Between September 1985 and August 1987, 405 patients with corneal ulceration were examined at Tribhuvan University Teaching Hospital in Kathmandu, Nepal. Males and females were equally affected. The most common predisposing cause of ulceration was corneal trauma, usually with organic agricultural materials. Microorganisms were grown from 324 (80%) of the ulcers. Pure bacterial cultures were obtained from 256 (63.2%) of the patients, whereas pure fungal cultures were obtained from 27 (6.7%) of the patients. In 41 patients (10.1%), corneal cultures yielded a mixed growth of bacteria and fungi. Of 68 positive fungal isolates obtained, 32 (47.0%) were identified as* Aspergillus* species.* Candida* species and* Fusarium* species were less commonly seen [20].

A 13-year study that was done in Paraguay from 1988 to 2001 and included 660 patients with infectious keratitis revealed that 79% of cases were culture positive of which 49% was fungal growth.* Aspergillus* species constituted 37% of fugal isolates less than* Fusarium* (41%) [21].

In a study done in Ghana in 1995, one or more organisms were cultured from 114 of 199 patients (57.3%), with the most common being* Fusarium* species,* Pseudomonas aeruginosa*, and* Staphylococcus epidermidis*. Fungi, alone or in combination, were isolated from 56% of the patients who had fungal growth, in total 122 patients (61.3%) [22].

This work reported that, among cases of mycotic keratitis, organic trauma was the most prevalent risk factor (38.2%), followed by ocular surgery in 32.7% of cases then the nonorganic trauma in 20.2% followed by the use of topical antibiotics in 6.1%, and systemic disease (such as diabetes) was the least frequent in only 2.2%. On the other hand, the nonorganic trauma was the most frequent risk factor for nonmycotic keratitis (45%) followed by the organic trauma (35%). Farmers were more exposed to organic trauma (with rice or maize stems or others) during farming so they were more exposed to mycotic keratitis. As regards the risk of ocular surgery among the cases, fungal contamination might occur preoperatively in those cases with negative cultures or postoperatively due to lowered body immunity (by the stressful surgery) and/or the use of a course of local and systemic corticosteroids with the surgery. In consistence with our findings, ocular trauma was the most frequent risk for either mycotic or nonmycotic keratitis in other studies [5, 15–22].

## 5. Recommendation

1. Community awareness of the risk factors.
2. Restriction of the abuse of topical corticosteroids or antibiotics.
3. Mycotic keratitis should be suspected in every patient with a corneal lesion, specially the resistant one, and should be ruled out before commencing steroids and antibiotics.
4. For a successful treatment, we need the following:
  - proper early clinical diagnosis,
  - proper laboratory diagnosis,
  - initiating a broad spectrum antifungal drug till laboratory findings prove the specific drug,
  - proper frequency of antifungal drugs,
  - proper timing for termination of therapy.

## Figures and Tables

**Table 1:** Age and sex distribution among mycotic and nonmycotic keratitis.

		Mycotic keratitis			Nonmycotic Keratitis		
		Male	Female	Total	Male	Female	Total
<40	No	50	24	74	72	30	102
	%	13.9	6.6	20.5	15.2	6.3	21.6
40–60	No	124	65	189	158	85	243
	%	34.3	18	52.4	65	35	51.4
>60	No	64	34	98	85	43	128
	%	17.7	9.4	27.1	18	9.1	27.1

Total	No	238	123	361	315	158	473
	%	65.9	34.1	100	66.6	33.4	100

The difference was insignificant in both age (*χ* ^2^ = 6.21, *P* = 0.258) and sex (*χ* ^2^ = 6.4, *P* = 0.739).

**Table 2:** Occupations among mycotic and nonmycotic keratitis.

	Mycotic keratitis		Nonmycotic keratitis	
	Number	%	Number	%
Occupation				
Professional and semiprofessional	8	2.2	94	19.9
Skilled workers	34	9.4	78	16.5
Unskilled workers	247	68.4	238	50.3
Housewives	72	20	63	13.3

Total	361	100	473	100

The difference was statistically significant (*χ* ^2^ = 21.41, *P* = 0.001).

**Table 3:** Family size and crowding index among mycotic and nonmycotic keratitis.

	Mycotic keratitis		Nonmycotic keratitis	
	Number	%	Number	%
Family size				
<4	72	20	173	36.6
4–6	151	41.8	204	43.1
>6	138	38.2	96	20.3
Crowding index				
<2	118	32.7	212	44.8
>2	243	67.3	261	55.2

The difference was statistically significant in case of family index (*χ* ^2^ = 10.67, *P* = 0.004) while it was insignificant in case of crowding index (*χ* ^2^ = 3.03, *P* = 0.081).

**Table 4:** Residence, water supply, and sewage disposal systems among mycotic and nonmycotic keratitis.

	Mycotic keratitis		Nonmycotic keratitis	
	Number	%	Number	%
Residence				
Rural	232	64.3	266	56.2
Urban	129	35.7	207	43.8
Water source				
Indoor	98	27.1	198	41.9
Outdoor	263	72.9	275	58.1
Sewage disposal				
Pipes	82	22.7	213	45
Conservancy system	279	77.3	260	55

The difference was not statistically significant in case of residence (*χ* ^2^ = 1.55, *P* = 0.212) while it was significant in both water supply (*χ* ^2^ = 4.95, *P* = 0.025) and sewage disposal (*χ* ^2^ = 10.78, *P* = 0.001).

**Table 5:** Time between the onset of complaints and examination among mycotic and nonmycotic keratitis.

	Mycotic keratitis		Nonmycotic keratitis	
	Number	%	Number	%
Time				
<7 days	222	61.5	402	85
7–14 days	58	16.1	71	15
14–30 days	81	22.4	0	0

The difference between the two groups was statistically significant as regards the different periods of time (*χ* ^2^ = 25.63, *P* = 0.3).

**Table 6:** Types of fungal infections among mycotic keratitis cases.

	Cases	
	Number	%
Type of fungus		
*Aspergillus flavus *	105	29.1
*Aspergillus niger *	58	16.1
*Mucorracemosus *	63	17.5
*Cladosporium sphaerospermum *	42	11.6
*Penicillium lanosum *	26	7.2
*Candida albicans *	11	3
Negative culture	56	15.5

Total	361	100

**Table 7:** Risk factors among mycotic and nonmycotic keratitis cases.

	Mycotic keratitis		Nonmycotic keratitis	
	Number	%	Number	%
Risk factors				
Organic trauma	138	38.2	213	45
Nonorganic trauma	73	20.2	142	30
Ocular surgery	118	32.7	0	0
Systemic disease	8	2.2	49	10.4
Prolonged local antibiotic	22	6.1	0	0
More than one factor	17	6	61	12.9
No obvious cause	2	0.6	69	14.6

Total	361	100	473	100
